# Supplementary material for: Assessment of minority frequency pretreatment HIV drug-resistant variants in pregnant women and associations with virologic non-suppression at term
Source: PLoS One. 2022 Sep 27;17(9):e0275254. doi: 10.1371/journal.pone.0275254 (PMC9514603; doi:10.1371/journal.pone.0275254)
Supplement: S1 Table — (DOCX) [file pone.0275254.s001.docx]

**S1 Table.** **Sequences of primers used for Illumina library preparation of samples.**

| Primer | Primer Sequences^a^ (5’ to 3’) | HXB2 Location |
| --- | --- | --- |
| cDNA | **CTCGGAGATGTGTATAAGAGACAG**NNNNNNNNAAYTTCTGTATACATTGACAGTCCA | 3303←3328 |
| Forward PCR | **TCGTCGGCAGCGTCAGATGTGTATAAGAGACAG**AAACAATGGCCATTRACAGAAGA | 2613→2635 |
| Reverse PCR | **GTCTCGTGGGCTCGGAGATGTGTATAAGAGACAG** | N/A |

^a^ Bold font indicates Illumina adapter sequence; HIV-specific portion of primer is denoted by regular font
